# Bacterial Communities Harboured by *Amblyomma Hebraeum* Infesting Small Stock in Mahikeng city, South Africa

**DOI:** 10.1007/s00248-025-02630-0

**Published:** 2025-11-08

**Authors:** Kealeboga Mileng, Sinalo Mani, Johannes J. Bezuidenhout, Prudent S. Mokgokong, Tsepo A. Ramatla, Oriel M. M. Thekisoe, Kgaugelo E. Lekota

**Affiliations:** 1 Unit for Environmental Sciences and Management, North-West University, Potchefstroom, 2520 South Africa; 2 Gastrointestinal Microbiology and Biotechnology, Agricultural Research Council Animal Production, Private Bag X2, Irene, 0062 South Africa; 3 Centre for Applied Food Safety and Biotechnology, Department of Life Sciences, Central University of Technology, 1 Park Road, Bloemfontein, 9300 South Africa

**Keywords:** *Amblyomma hebraeum*, *16S rRNA*, Bacterial communities, Next-generation sequencing, Tick-borne pathogens

## Abstract

Ticks are important vectors of pathogens affecting livestock productivity and public health, yet their bacterial communities remain poorly characterized in many parts of South Africa. This study investigated the bacterial diversity and potential pathogenic bacterial etiology associated with *Amblyomma hebraeum* ticks collected from sheep and goats in Mahikeng, North West province. A total of 168 adult ticks were sampled across four villages. Microbiome profiling was performed using high-throughput sequencing of the V3–V4 hypervariable regions of the 16*S rRNA* gene on the Illumina MiSeq platform. High-throughput 16S *rRNA* sequencing revealed 16,193 ASVs in goat-derived ticks and 16,510 ASVs in those from sheep. Proteobacteria emerged as the dominant phylum across all samples, with ticks collected from goats showing a particularly high dominance of *Rickettsia* spp. (51.64% relative abundance), suggesting potential zoonotic risks. In contrast, ticks from sheep harboured significantly more diverse and evenly distributed bacterial communities, as indicated by Shannon (*p* = 0.0138) and Simpson (*p* = 0.0233) diversity indices, despite comparable species richness. A core microbiome comprising 1,374 ASVs (32.3%) was shared across all ticks, alongside 1,504 and 1,372 unique ASVs in goat- and sheep-derived ticks, respectively. Notably, several medically and veterinary-relevant genera, including *Coxiella*, *Ehrlichia*, *Staphylococcus*, *Bacillus*, *Acinetobacter*, *Corynebacterium*, and *Streptococcus,* were detected across both host groups. While total species richness was comparable between hosts, alpha diversity indices that account for evenness revealed host-based differences, and beta diversity patterns further showed clear separation of bacterial communities by host species. This study indicates that the host plays a crucial role as an ecological driver affecting the diversity of microbial communities associated with ticks. This study improves our understanding of the diversity, composition, and abundance of tick-associated microbiomes and pathogens in South African small ruminants. These insights support the development of microbiome-targeted strategies for detecting and controlling tick-borne diseases.

**Supplementary Information:**

The online version contains supplementary material available at 10.1007/s00248-025-02630-0.

## Introduction

Ticks are known to harbour diverse microorganisms, which threaten the health of animals and humans [1]. These ectoparasites can negatively affect livestock health and productivity, mainly by transmitting several zoonotic and livestock pathogens [2]. Among the different tick species distributed in Southern Africa, *Amblyomma hebraeum* ticks are a key vector of *Ehrlichia ruminantium* [3, 4]. *Ehrlichia ruminantium* is the causative agent of heartwater, a fatal rickettsial disease affecting ruminants, which poses a significant threat to the livestock industry [5, 6]. This disease seriously threatens small-stock production in rural communities. The villages around Mahikeng, North West province, are particularly at risk due to reliance on small ruminants for income and food security [7]. Additionally, *A. hebraeum* is known to host a variety of bacteria, including symbionts, commensals, and potential pathogens that may influence the tick's physiology, vector competence, and disease transmission ability [8].

Microbial communities found in ticks include vast amounts of symbionts and putative pathogens in addition to their role as vectors for other known pathogens [9]. The mixed bacterial communities present in ticks have the potential to alter the biological and epidemiological characteristics of ticks, as well as their capacity to transmit tick-borne diseases [10]. Despite the microbiome's involvement in tick-pathogen interactions being increasingly established, the microbiome of *A. hebraeum* has not been the focus of many studies, especially not in small-stock farming regions of rural South Africa. Knowledge of these bacterial communities can enhance our understanding of the microbial ecology within ticks, highlighting how ticks serve as microecosystems that may facilitate the emergence of new pathogens [11].

In South Africa, recent studies have investigated bacterial communities in *A. hebraeum* ticks. Rickettsial species are commonly associated with African tick bite fever. *Rickettsia africae* was isolated from *A. hebraeum* ticks inhabiting livestock in the North West province of South Africa [12]. From the same geographical area, several *E. ruminantium* variants were discovered in *A. hebraeum* ticks, emphasizing the genetic heterogeneity of this heartwater pathogen [7]. High-throughput sequencing revealed the differential abundance of potentially pathogenic and endosymbiotic bacteria in *Amblyomma* ticks, including *Rickettsia*, *Escherichia*, and *Coxiellaceae* [13]. In addition, several off-target antibiotic resistance markers were in the tick microbiome, which affects disease management [13]. These findings contribute to understanding tick-associated bacterial communities and their potential impact on pathogen transmission and treatment strategies. Investigating tick microbiomes can uncover potential symbiotic relationships and identify bacteria that might influence tick physiology and pathogen transmission.

Mahikeng, located in the North West Province of South Africa, is a vital livestock farming region where small stock, such as goats and sheep, are commonly raised. The presence of *A. hebraeum* in these villages raises concerns regarding its potential to harbour and transmit bacterial pathogens. However, studies focusing on the bacterial diversity within *A. hebraeum* from small stock in this region remain limited. This study investigated the bacterial communities harboured by *A. hebraeum* collected in sheep and goats from Mahikeng using high-throughput 16S *rRNA* metabarcoding. This study utilized molecular techniques to identify potential pathogenic and symbiotic bacteria, enhancing our comprehension of tick-borne microbial ecology within the South African context. The findings may guide future vector control efforts and inform disease management strategies.

## Materials and Methods

### Study Area and Sample Collection

**Fig. 1:**
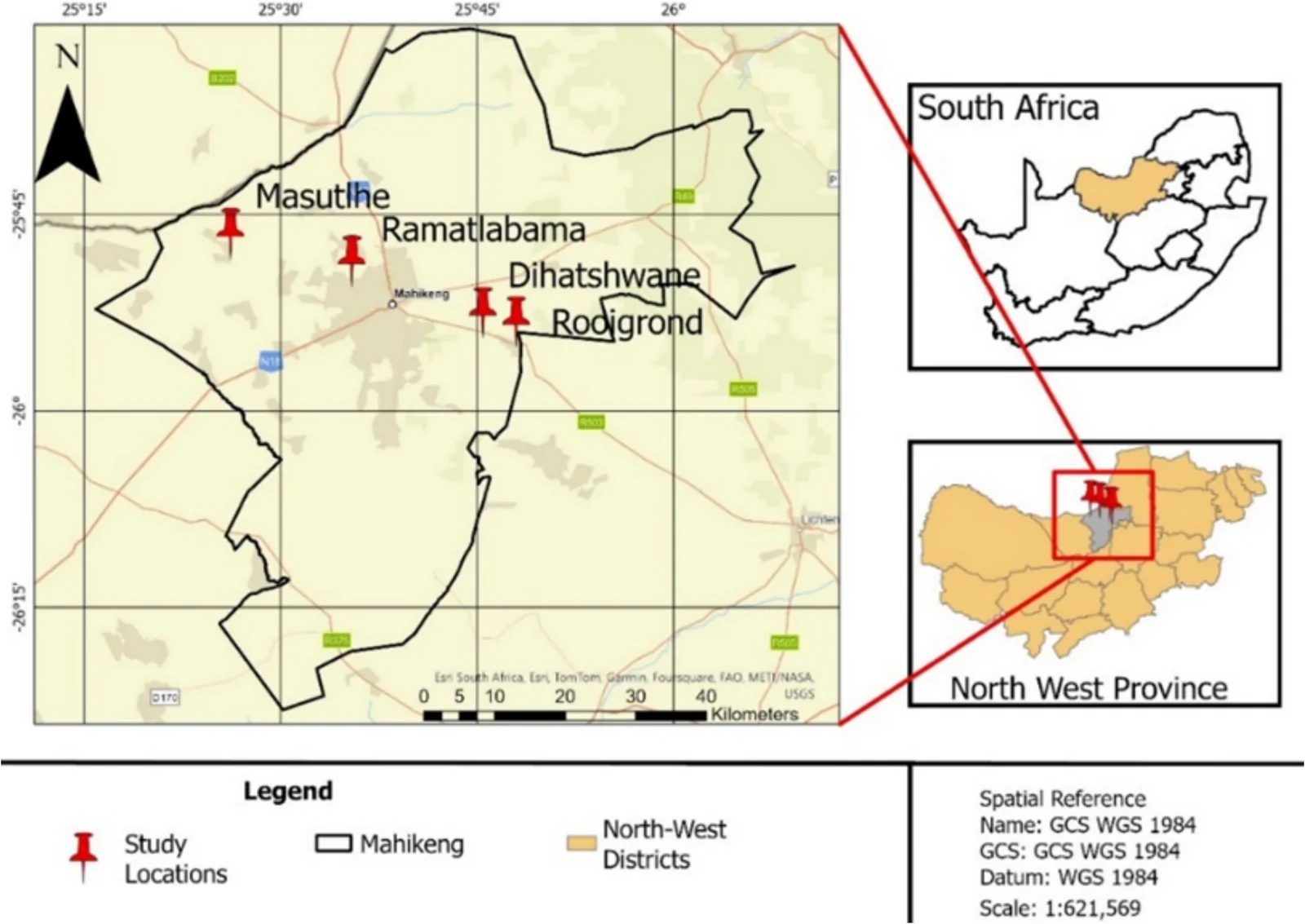
A map indicating the location of Masutlhe, Ramatlabama, Dihatshwane, and Rooigrond in Mahikeng. The study areas are marked with red point markers

Tick specimens were collected (February 2024) in villages around four regions: Masutlhe (25°48′12.0"S, 25°24′50.1"E), Ramatlabama (25°42′06.0"S, 25°40′40.9"E), Dihatshwane (25°54′25.6"S, 25°45′12.7"E) and Rooigrond (25°55′18.3"S, 25°47′58.7"E) within the Mahikeng city of North West province, South Africa (Fig. 1). The region is characterized by mixed farming systems with strong reliance on small stock. During routine farm visits, 168 *Amblyomma hebraeum* ticks were collected from infested goats and sheep. Ticks were collected and preserved in tubes containing 70–90% ethanol for storage.

### Tick Identification

Morphological identification of ticks to the species level was performed using a Nikon SMZ745 stereo microscope and relevant identification keys [14]. Furthermore, the genetic identification was used to supplement morphology by PCR amplification and sequencing of the *16S rRNA* and *18S rRNA* nuclear genes [15] and aligning them on BLASTn for identity matches.

### DNA Extraction of Amblyomma Hebraeum

Thirty (n = 30) tick pools were created, each comprising an average of 3- ticks, classified by collection site and host species. The pools consisted of 15 derived from goats and 15 from sheep, totalling 168 collected ticks. Prior to DNA extraction, ticks were washed twice with 70% ethanol and distilled water and allowed to air dry on sterile paper. Each sample was cut with sterile scalpels in a 1.5 ml microcentrifuge tube. DNA was extracted from the ticks using QIAGEN DNeasy® Blood & Tissue Kit (Qiagen, Hilden, Germany) according to the manufacturer’s instructions. DNA integrity and concentration were assessed using agarose gel electrophoresis and fluorometric quantification with Qubit 2.0 (Invitrogen, Carlsbad, CA, USA). The eluted DNA was subsequently stored at −20 °C for further analysis.

### Genetic Identification of Ticks Using 16S rRNA and 18S rRNA PCR Assays

Genetic identification of ticks was performed using polymerase chain reaction (PCR) targeting the mitochondrial *16S rRNA* gene with primers 16S-F (5'-CTG CTC AAT GAT TTT TTA AAT TGC TGT GG-3') and 16S-R (5'-CCG GTC TGA ACT CAG ATC AAG T-3'), as well as the nuclear *18S rRNA* gene with primers 18S-F (5'-CATTAAATCAGTTATGGTTCC-3') and 18S-R (5'-CGCCGCAATACGAATGC-3') [15]. Each PCR reaction consisted of a total reaction of 25 μL consisting of 12.5 μL of a 2X DreamTaq Green Mastermix (0.4 mM dATP, 0.4 mM dCTP, 0.4 mM dGTP, 0.4 mM dTTP, 4 mM MgCl2, and loading buffer) (ThermoFisher Scientific, South Africa), 8.5 μL of nuclease-free water, 2 μL of the template DNA, and 1 μL of each oligonucleotide primer. PCR conditions were as follows: one step of denaturation at 94 °C for 2 min; 35 cycles of denaturation at 94 °C for 30 s., annealing at (16S rRNA: 72 °C; 18S rRNA: 72 °C) for each gene assay for 30 s., and elongation at 68 °C for 2 min.; and one step of final elongation at 68 °C for 5 min. The PCR amplification products were confirmed by electrophoresis on a 1.5% agarose gel, stained with ethidium bromide (EtBr), and visualized under UV light.

### Library Preparation and Sequencing

Genomic DNA extracted from *A. hebraeum* ticks was used to amplify the V3–V4 hypervariable regions of the bacterial *16S rRNA* gene, using primers 341 F and 805R with Illumina overhang adapters [16]. A negative control containing only elution buffer was included in the PCR assays to monitor potential contamination. Amplicons were generated using KAPA HiFi HotStart ReadyMix (Kapa Biosystems, Wilmington, MA, USA) and purified with AMPure XP magnetic beads (Beckman Coulter Genomics, USA). Dual indices were added in a second PCR step using the Nextera XT Index Kit v2, followed by another round of purification. Libraries were sequenced on an Illumina MiSeq (2 × 300 bp) using the v3 kit at the North-West University Sequencing platform in Potchefstroom, South Africa.

### Bioinformatics and Statistical Analysis

DADA2 was used to process reads obtained from the sequencing platform. Raw reads were first demultiplexed, and primers and indexes were removed [17], followed by quality filtering and chimera removal. Reads were truncated at 250 bp with a maximum expected error of 5, allowing no ambiguous bases. Error rates for forward and reverse reads were learned independently and then merged. The amplicon sequence variant table was constructed, and taxonomy classification was assigned to the sequence variants using SILVA 138.1 database up to genus level. The sequence data were processed with DADA2 amplicon sequence variants (ASVs).

Downstream analyses and visualisation were done using the *phyloseq* package in R. Alpha diversity was assessed using Observed ASVs, Chao1, Shannon, and Simpson indices. Beta diversity was evaluated using Bray–Curtis dissimilarity and Jaccard distance matrices, visualized through Principal Coordinate Analysis (PCoA) and Non-Metric Multidimensional Scaling (NMDS), respectively. Microbial abundance was visualised using *ggplot2.* The statistical significance of alpha diversity differences between host groups was tested using the Kruskal–Wallis test. Adonis tests were used to assess beta diversity differences for host groups and for the locations.

## Results

### Tick Species Identification

Tick specimens were morphologically identified as *Amblyomma hebraeum*. Molecular confirmation was achieved through amplification and sequencing of the *16S rRNA* and 1*8S rRNA* genes. BLASTn analysis of the resulting sequences showed high similarity to reference sequences in GenBank, with identities ranging from 99.27% to 100% for 16S *rRNA* and 99.36% to 99.87% for 18S *rRNA*. The GenBank accession numbers for the 16S rRNA gene sequences are: PP80989, PP809810, PP809812, PP809813, PP809811, PP809814, PP809815, PP809816, PP809817, PP809818, PP809824, and PP809819. The corresponding accession numbers for the 18S rRNA gene sequences are: PP826352, PP830056, PP830059, PP830060, PP830061, PP830062, PP830064, PP842196, and PP830071.

### Alpha-diversity Associated with Amblyomma Hebraeum Ticks from Sheep and Goats

Illumina MiSeq sequencing generated a total of 528,482 raw sequence reads from *A. hebraeum* ticks in the goat dataset and 338,497 reads from the sheep dataset. After trimming, quality-checking, and removing chimeric sequences, as well as low-quality and non-target amplicons, the datasets were reduced to 409,037 valid reads for the goat samples and 215,319 valid reads for the sheep samples. These reads were then clustered into 16,193 amplicon sequence variants (ASVs) for the goat dataset and 16,510 ASVs for the sheep dataset.

Diversity rarefaction curves were generated using amplicon sequence variants (ASVs) to assess the sequencing depth and microbial diversity within the microbiomes of *A. hebraeum* ticks collected from goats and sheep. This analysis was based on the 16S *rRNA* amplicon sequencing data. The rarefaction curves (Supplementary Figure S1) display species richness across varying sequence sample sizes, reflecting the diversity of bacterial communities associated with *A. hebraeum* ticks. Ticks collected from goats (represented by red curves) reached a plateau in species richness at lower sequencing depths than sheep ticks (depicted by teal curves). This indicates that the microbial diversity associated with ticks from goats is less complex and reaches saturation faster than sheep. In contrast, ticks collected from sheep required a greater sequencing depth to capture the full diversity of their microbiome, as their curves show a slower progression towards a plateau.

**Fig. 2:**
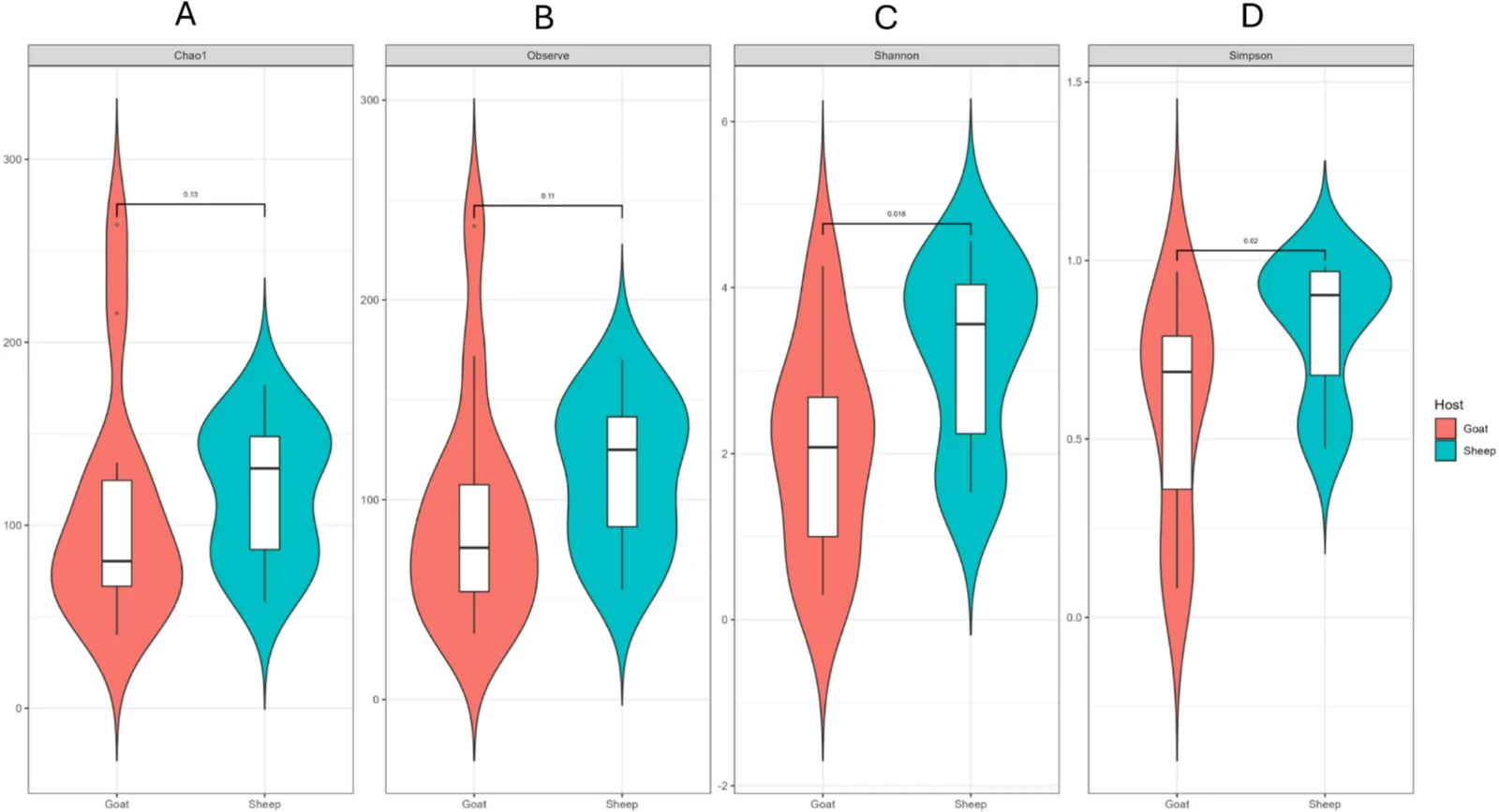
Alpha diversity analysis of the microbial communities associated with *Amblyomma* *hebraeum* ticks collected from sheep and goats. **A** Chao1, **B** Observed ASVs, **C** Shannon diversity index and (**D**) Simpson diversity index richness estimator. Significant differences were observed for Shannon and Simpson indices (*p* < 0.05), indicating higher microbial diversity and evenness in ticks from sheep

Alpha diversity was assessed using richness estimators and diversity indices: Observed ASVs and Chao1 (richness estimators) and Shannon and Simpson indices (diversity and evenness metrics). Statistical comparison revealed distinct patterns in microbial diversity influenced by host species (Fig. 2 A-D). The Kruskal–Wallis test was used to evaluate the significance. For richness, both Chao1(A) and Observed ASVs (B) showed no significant differences (*p* = 0.13 and *p* = 0.11, respectively), suggesting that the number of bacterial taxa, including rare species, was comparable between the two host-associated tick populations. In contrast, significant differences were observed for diversity indices. The Shannon(C) index (*p* = 0.018) and the Simpson(D) index (*p* = 0.02) were both significantly higher in ticks collected from sheep, indicating that these ticks harbored microbial communities that were not only more diverse but also more evenly distributed. These results demonstrate that while microbial richness was similar between hosts, ticks from sheep supported greater microbial diversity and evenness compared to those from goats, highlighting a host-associated influence on the structure of tick microbiomes.

Additionally the alpha diversity of bacterial communities associated with *Amblyomma hebraeum* ticks was evaluated across four collection sites (Dihatshwane, Masutlhe, Ramatlabama, and Rooigrond) using richness estimators (Observed ASVs, Chao1) and diversity indices (Shannon, Simpson) (Supplementary Figure S2 A–D). The Kruskal–Wallis test was applied to assess statistical significance between locations. For richness estimator (Chao1(A) and Observed ASVs(B)), no statistically significant differences were observed between locations (all *p* > 0.05). This indicates that the overall number of bacterial taxa, including rare species, was broadly comparable among ticks collected from different sites. In terms of diversity and evenness, the Shannon(C) and Simpson(D) indices also did not show statistically significant differences across locations (all *p* > 0.05). While some variability in median values was evident, ticks from Masutlhe and Ramatlabama tended to show slightly higher Shannon and Simpson diversity compared to those from Dihatshwane and these trends were not statistically robust.

### Beta Diversity Estimate of Bacterial Communities Associated with Amblyomma Hebraeum Ticks

**Fig. 3:**
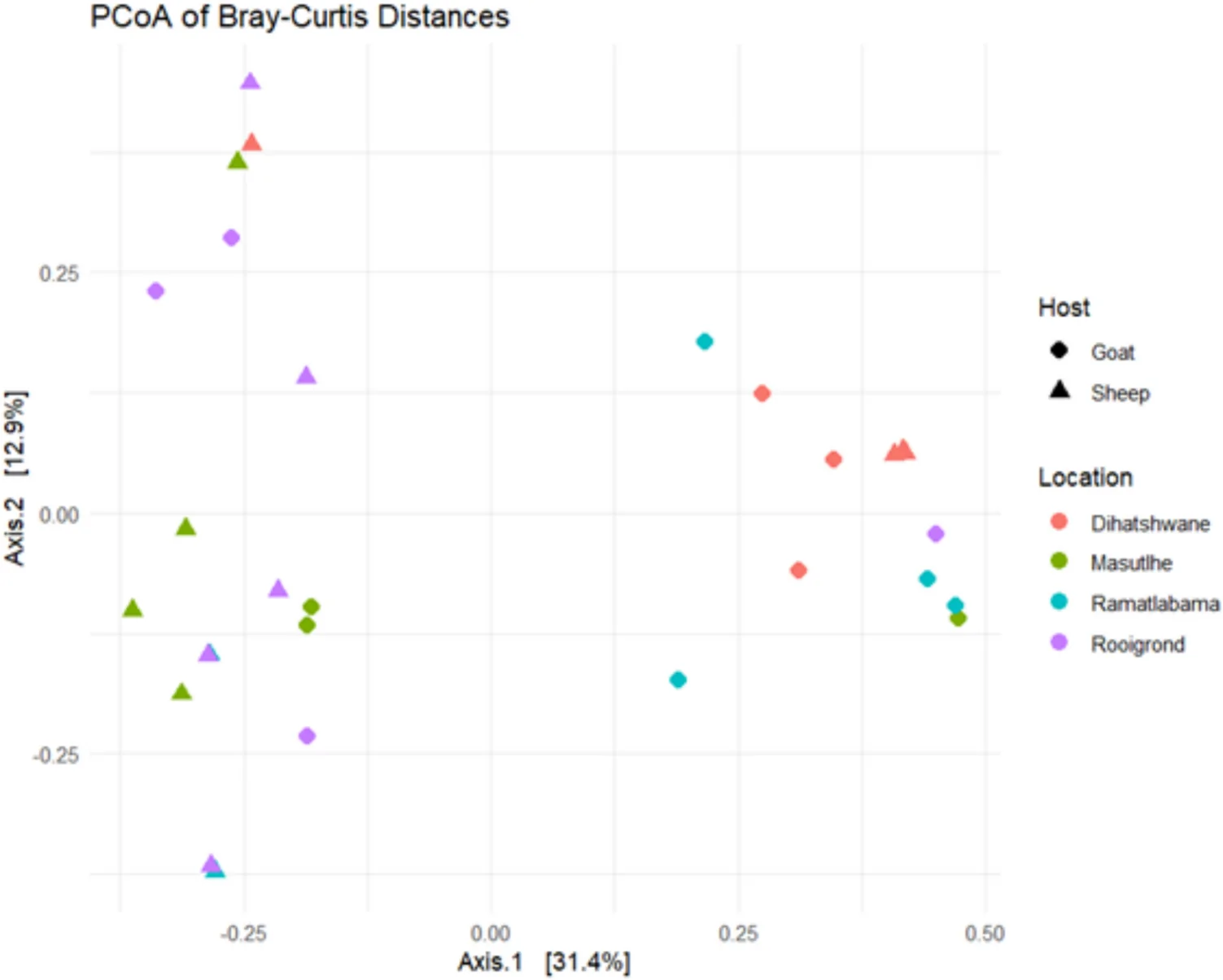
Principal Coordinates Analysis (PCoA) based on Bray–Curtis distances showing bacterial community composition of *Amblyomma hebraeum* ticks collected from sheep and goats across four villages in Mahikeng

The Principal Coordinate Analysis (PCoA) plot, based on Bray–Curtis dissimilarity, visualizes the differences in bacterial community structure in *A. hebraeum* ticks collected from goats and sheep and locations (Fig. 3). Each point represents the entire microbial profile of a single tick. Samples in closer proximity share more similar bacterial communities than those further apart. The plot reveals moderate overlap between the groups defined by tick host species (sheep vs. goat) and collection location (Dihatshwane, Masutlhe, Ramatlabama, and Rooigrond). This overlap is common in microbial ecology and indicates that, while these factors are significant, a substantial variation arises from within-group differences or other unmeasured variables, such as individual animal health, tick life stage, or environmental micro-niches. Despite this overlap, a visual inspection shows a discernible tendency for points of the same colour, particularly for the different villages, to cluster more closely together. Permutational multivariate analysis of variance (PERMANOVA) confirmed that both the host animal (sheep or goat) (R2 = 0.09, F = 2.64, *p* = 0.01) and the collection village (R2 = 0.19, F = 1.97, *p* = 0.005) were significant predictors of microbial community composition. Analysis of similarities (ANOSIM) further supported these findings.

The bacterial community structures between *A. hebraeum* ticks from different host species were compared. An ANOSIM confirmed a statistically significant, albeit weak (Supplementary Figure S3), separation between the microbiomes of ticks collected from goats versus sheep (R = 0.163, *p* = 0.014). ANOSIM also confirmed a significant separation between locations (Supplementary Figure S4), though the effect size was weak (R = 0.107, *p* = 0.047). This suggests that while the central tendency of microbial communities differs significantly between villages, there is considerable within-village variation and overlap between them.

### The Bacterial Composition at the Phylum Level

**Fig. 4:**
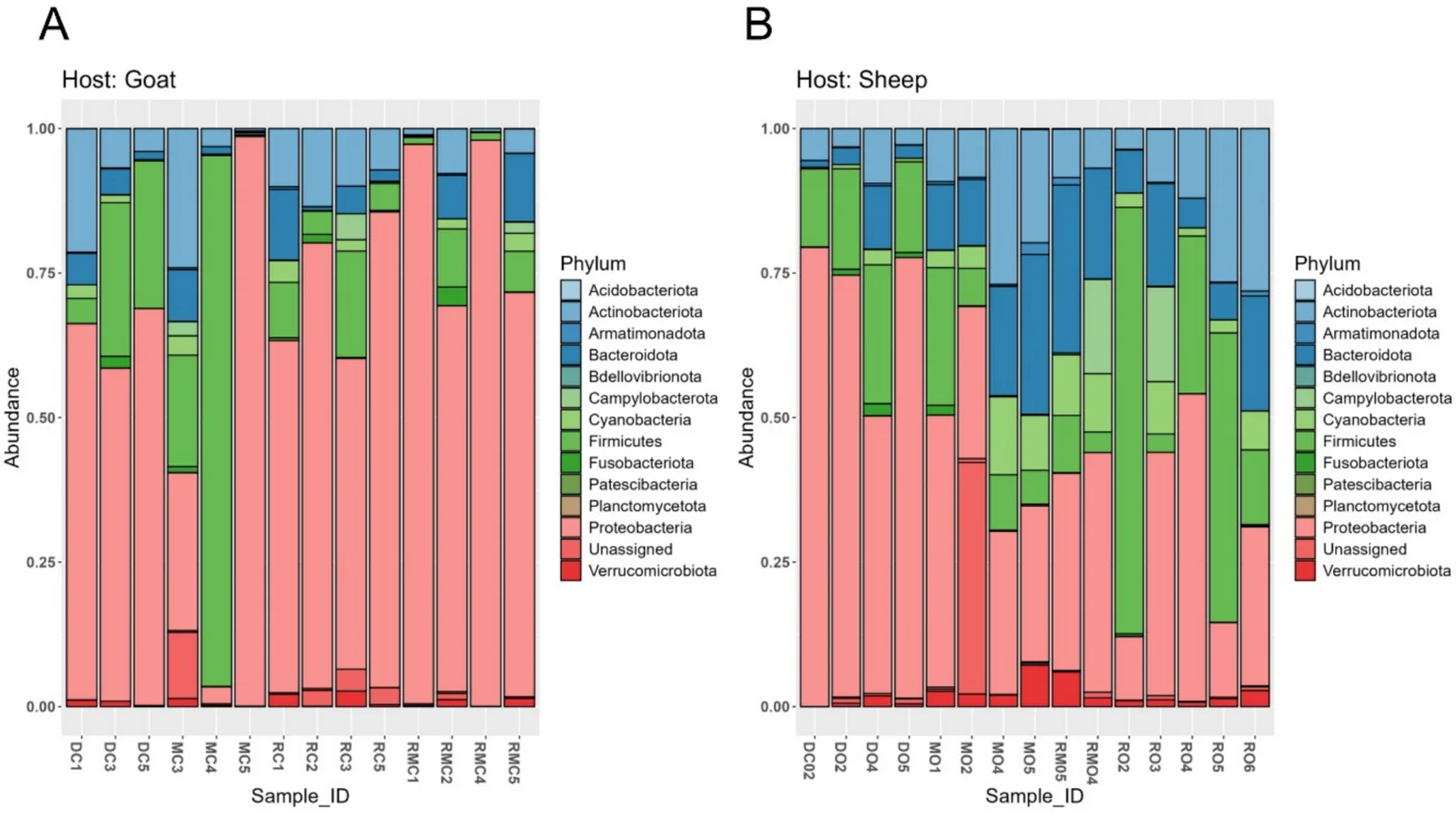
Stacked bar plot showing the relative abundance of bacterial phyla identified in *Amblyomma hebraeum* ticks collected from (**A**) goats (*n* = 15) and (**B**) sheep (*n* = 15) pools. The dominant bacterial families in goat-derived ticks included *Rickettsiaceae* (50.68%), followed by *Bacillaceae* (9.81%), *Coxiellaceae* (8.85%), *Staphylococcaceae *(6.71%) and *Corynebacteriaceae *(4.98%). The least abundant families in ticks included* Xanthomonadaceae*, *Sphingomonadaceae*, and *Cyclobacteriaceae *(~0.3–0.6%) as shown in Supplementary Figure S5A. In sheep-derived ticks, the dominant families were *Rickettsiaceae *(18.34%) followed by *Staphylococcaceae* (12.98%), *Coxiellaceae *(7.57%), *Bacillaceae* (7.47%) and *Comamonadaceae* (5.46%). The least abundant families included* Intrasporangiaceae*, *Saprospiraceae*, and *Cyclobacteriaceae* (~1.2–1.95 %), depicted in Supplementary Figure S5B

The taxonomic abundances of all ASVs at the phylum level were visualised using a stacked bar plot based on relative abundances (Fig. 4). A total of 13 bacterial phyla were assigned to all *A. hebraeum* ticks from sheep and goats. In ticks from goats, the five most abundant bacterial phyla were Proteobacteria (66.12%), followed by Firmicutes (16.01%), Actinobacteriota (8.16%), Bacteroidota (4.36%), and Cyanobacteria (1.32%). The least abundant bacterial phyla included Planctomycetota, Acidobacteriota, and Bdellovibrionota (~ 0.002–0.02%), as shown in Fig. 4A. In ticks from sheep, the five most abundant bacterial phyla in *A. hebraeum* ticks were Proteobacteria (41.80%), followed by Firmicutes (19.80%), Bacteroidota (12.76%), Actinobacteriota (11.99%) and Cyanobacteria (5.09%). The remaining bacterial phyla had relatively low abundances (< 1%). The least abundant bacterial phyla were Patescibacteria, Planctomycetota, and Acidobacteriota (~ 0.02–0.04%) depicted in Fig. 4B.

**Fig. 5:**
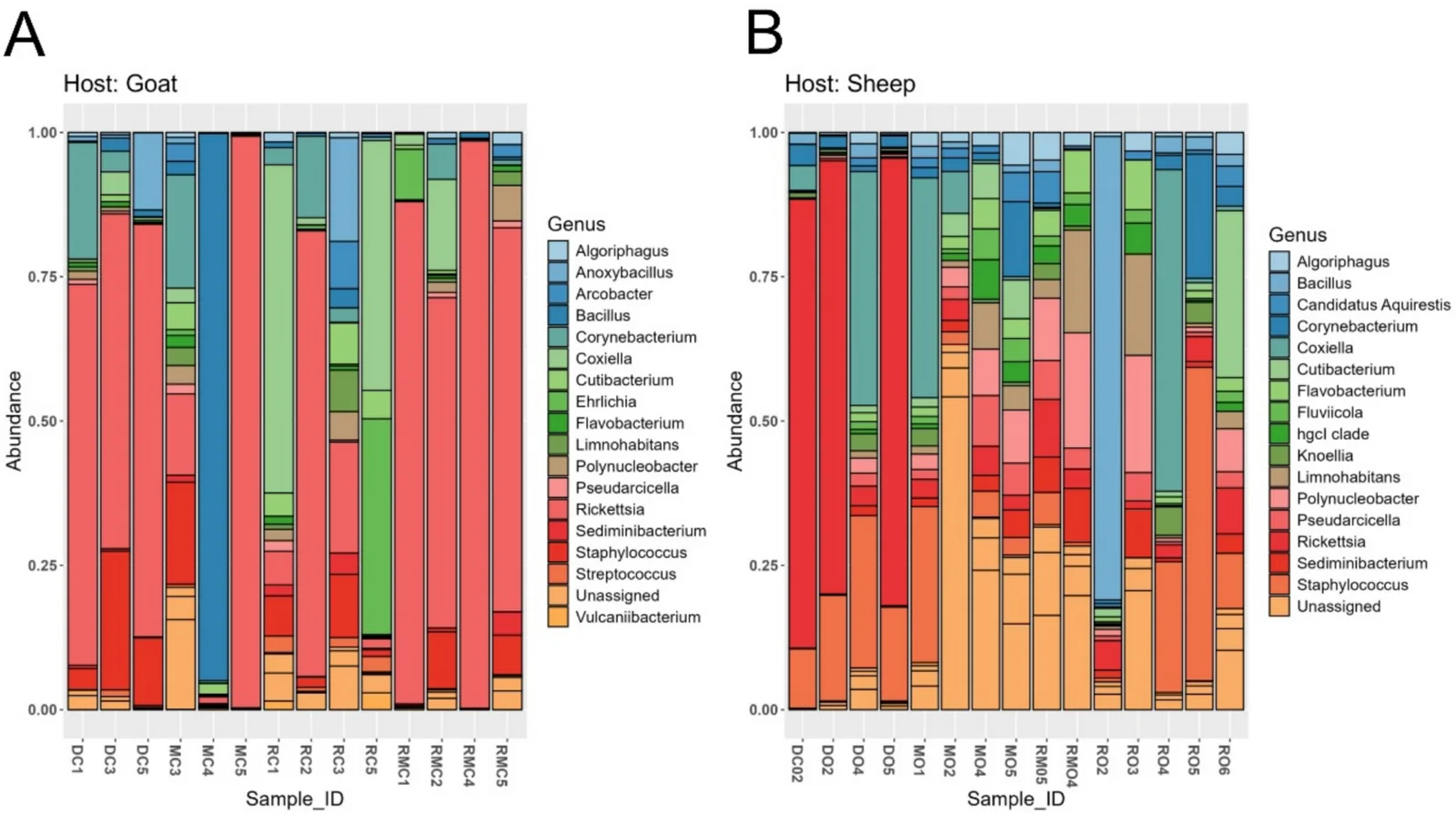
Stacked bar plot represents the relative abundance of bacterial genera in *Amblyomma hebraeum* ticks from (**A**) goats (**A**) goats (*n* = 15) and (**B**) sheep (*n* = 15) pools

At the genus level, the microbiota composition in ticks from goats revealed the following dominant genera: *Rickettsia* (51.64%), followed by *Coxiella* (9.01%), *Bacillus* (7.77%), *Staphylococcus* (6.83%), and *Corynebacterium* (5.08%) (Fig. 5 A). The least abundant genera included *Vulcaniibacterium* (0.31%), *Pseudarcicella* (0.54%), and *Algoriphagus* (0.57%). In contrast, the microbiota composition in ticks from sheep showed a slightly different distribution: *Rickettsia* (18.85%) was the most predominant, followed by *Staphylococcus* (13.44%), *Coxiella* (9.97%), *Bacillus* (6.55%), and *Polynucleobacter* (5.88%), depicted in Fig. 5B. The least abundant genera among sheep-derived ticks included *Knoellia*, *Fluviicola*, and *Candidatus Aquirestis* (~ 1.35–1.61%).

### Host-based Differences in Microbial Genera

**Fig. 6:**
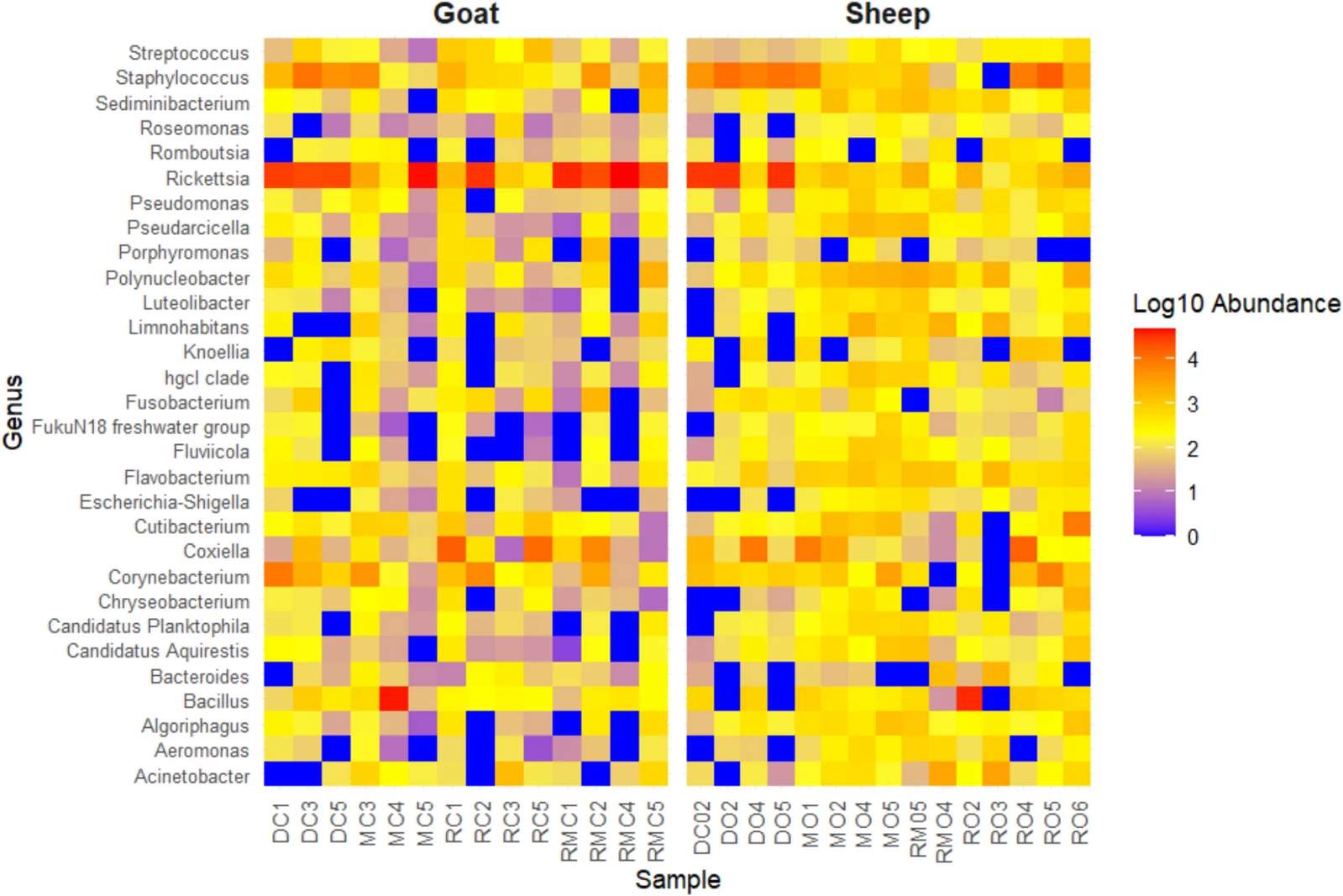
Heatmap of the top 30 most abundant bacterial genera in *Amblyomma hebraeum* ticks collected from sheep and goats. Relative abundances were log10-transformed to enhance the visualization of differences. Relative values for each genus are depicted by the colour intensity according to the legend on a scale of 0 to 4; warmer colours indicate higher abundance. The heatmap is clustered by host, with goat and sheep tick samples grouped accordingly

The heatmap illustrates the relative abundance (log10-transformed) of the top 30 bacterial genera across *A. hebraeum* ticks collected from goats and sheep (Fig. 6), revealing distinct host-specific patterns in microbial composition. Specific genera, such as *Rickettsia*, *Streptococcus*, and *Staphylococcus*, exhibit higher relative abundance in ticks from goats, as indicated by the warmer colours (red and orange). This is consistent with the relative abundance observed in Fig. 5. In contrast, *Pseudomonas* and *Roseomonas* spp. appear more prominent in ticks from sheep. Many genera are present in both host-associated communities (e.g., *Bacillus*, *Flavobacterium*, *Escherichia-Shigella*), though their relative abundances differ by host.

In contrast, low-abundance genera such as *Corynebacterium*, *Cutibacterium*, and *Chryseobacterium* spp. are indicated by cooler colours (blue), likely representing fewer dominant members of the microbiota. In the heatmap, genera such as *Rickettsia*, *Streptococcus*, and *Staphylococcus* were more enriched in goat ticks, consistent with the relative abundance profiles. Notably, *Streptococcus* appeared in goat ticks but was not among the top five genera in sheep, suggesting its presence was host-associated but more prominent in goats.

### Shared and Unique Bacterial ASVs Across Hosts and Locations

**Fig. 7:**
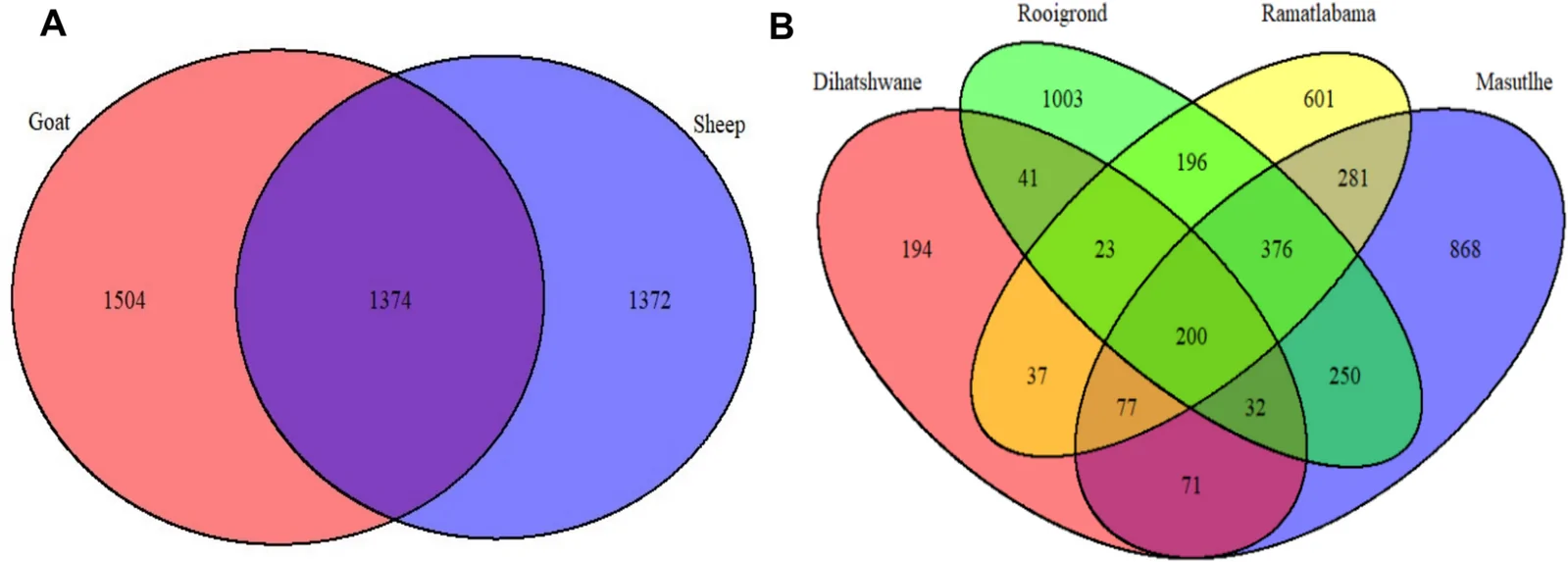
Venn diagrams showing the shared and unique ASVs in *Amblyomma*. **A** Distribution of genera ASVs between ticks collected from sheep and goats. **B** Distribution of ASVs across four sampling locations among four villages: Dihatshwane, Rooigrond, Ramatlabama, and Masutlhe

Venn diagrams of the bacterial ASVs were constructed to determine the shared species richness between *A. hebraeum* ticks collected from different hosts and locations (Fig. 7). The Venn diagram reveals the distribution of bacterial ASVs found in *A. hebraeum* ticks collected from goats and sheep. Figure 7 A shows that 1,374 ASVs are shared between ticks from goats and sheep, with 1,504 unique to goats and 1,372 unique to sheep. These unique ASVs may reflect host-specific influences such as differences in diet, immune responses, or physiological conditions. Overall, the combined richness of 4,250 bacterial ASVs highlights the diversity of microbial communities associated with *A. hebraeum* ticks in these hosts.

The second Venn diagram illustrates the distribution of bacterial ASVs found in *A. hebraeum* ticks collected from four locations: Dihatshwane, Rooigrond, Ramtlabama, and Masutlhe depicted in Fig. 7B. The central region shows that 200 bacterial ASVs are shared across all four locations, indicating a core microbiome that persists regardless of geographic differences. Beyond this shared core, specific overlaps highlight further relationships. For example, 376 bacterial ASVs are shared between Rooigrond and Ramtlabama, while 281 are shared between Ramtlabama and Masutlhe. Unique bacterial ASVs were observed in each location, with Dihatshwane, Rooigrond, Ramtlabama, and Masutlhe harbouring 194, 1,003, 601, and 868 exclusive ASVs.

## Discussion

Bacterial communities were investigated in *Amblyomma hebraeum* ticks collected from sheep and goats in Mahikeng villages, North West Province, South Africa. Understanding livestock health, tick-borne pathogen transmission, and the dynamics of tick-borne diseases requires understanding the tick microbiome in sheep and goats. Despite the significance, studies on the tick microbiome of South African small stock are scarce. The study revealed a significant divergence in bacterial communities associated with *A. hebraeum* ticks from sheep and goats, highlighting a host-dependent microbial diversity.

The alpha diversity analyses, based on Observed ASVs, Shannon, Simpson, and Chao1 indices, revealed key differences in microbial community structure of the two host species. The Shannon index (*p* = 0.018) and the Simpson index (*p* = 0.02) were significantly higher in ticks collected from sheep, indicating that sheep-associated ticks hosted more diverse and evenly distributed microbiota than goat-associated ticks. The absence of significant differences in species richness, coupled with the marked variation in evenness, suggests that ecological determinants may shape a hierarchical organization of the microbial community, where particular species assume dominant roles. The Shannon and Simpson indices are particularly relevant in assessing microbial diversity as they account for species richness and evenness, providing a more comprehensive understanding of community structure [18]. In contrast, the Observed ASVs and Chao1 indices, which did not show significant differences (*p* > 0.05), suggest that while the diversity of microbial species may differ, the overall richness of species present in ticks from both hosts remains comparable. This distinction is critical as it implies that while the composition of the microbial community varies, the number of species may not be a limiting factor in the ecological interactions occurring within these tick populations [19].

The beta diversity analysis of *A. hebraeum* ticks revealed a moderate degree of overlap between the groups defined by tick host species (sheep vs. goat) and collection location (Dihatshwane, Masutlhe, Ramatlabama, and Rooigrond). These findings suggest that host species strongly influence tick microbiota composition, likely due to differences in skin characteristics, resident microbiomes, immune responses, or environmental exposures encountered during feeding [20]. Additionally, they may have significant consequences for vector control strategies to mitigate tick-borne diseases. The differences in microbial community structures could influence the vector competency of *A. hebraeum*, as specific microbial taxa are known to affect tick physiology and pathogen transmission [21, 22]. For instance, particular endosymbionts can enhance ticks' survival and reproductive success, affecting their role as vectors for pathogens [23, 24]. Understanding these dynamics is essential for developing targeted interventions that could disrupt the transmission of tick-borne pathogens by manipulating the tick microbiome [25].

At the phylum level, Proteobacteria were the dominant in ticks from both hosts, accounting for 66.12% in goats and 41.80% in sheep. The predominance of Proteobacteria in various tick species has been consistently documented, suggesting its critical role in the microbial communities associated with these ectoparasites [26]. Recent studies on tick microbiota across multiple species reveal Proteobacteria as the predominant phylum, followed by Bacteroidete*s* and Firmicutes [27, 28]. The predominance of Proteobacteria has also been reported in the ticks from the AI Khotha region of Saudi Arabia [29]. These findings suggest a universal trend in tick microbiota composition. This widespread dominance may be attributed to the extensive phylogenetic breadth of the phylum, which encompasses metabolically versatile taxa capable of surviving diverse environmental conditions and host-associated microhabitats [30]. Such functional plasticity likely provides adaptive advantages, allowing Proteobacteria to persist through fluctuating resource availability, oxidative stress, and immune pressures within the tick or host environment. Consequently, the abundance of Proteobacteria in goat- and sheep-associated ticks reflects its ecological resilience and may underscore its role in shaping tick physiology, development, and vector competence [31, 32].

At the family level, *Rickettsiaceae*, *Staphylococcaceae*, *Coxiellaceae*, and *Bacillaceae* were dominant across host species, but their relative abundances varied. Rickettsiaceae strongly dominated goat-derived ticks (50.68%), whereas sheep-derived ticks exhibited a more balanced representation of families. This suggests host-specific enrichment with potential functional implications for microbial interactions. It also reflects the alpha diversity results: microbial communities in sheep ticks were more evenly distributed among taxa, whereas a few specific groups, such as Rickettsiaceae, dominated those in goat ticks. Although minor in abundance, low-abundance families such as *Saprospiraceae*, *Cyclobacteriaceae*, and *Intrasporangiaceae* may contribute to ecological balance and microbial succession [33].

Furthermore, at the genus level, *Rickettsia* dominated in *A. hebraeum* ticks from both hosts, although their relative abundances differed. In the *A. hebraeum* ticks from goats, *Rickettsia* accounted for 51.64%, whereas ticks from sheep showed 18.85%. The relative abundance of *Rickettsia* spp. in ticks from goats indicates that goats may serve as significant reservoirs for *Rickettsia* spp. This aligns with findings from previous studies that have reported higher tick burdens and associated pathogen prevalence in goats compared to sheep [34, 35]. The presence of *Rickettsia* spp. in these ticks raises public health concerns, such as rickettsial infections, which can be transmitted through tick bites, causing various human diseases, including spotted fever rickettsiosis [36, 37].

In addition, the top 30 genera heatmap detected pathogenic genera including *Coxiella, Bacillus, Staphylococcus, Corynebacterium, Ehrlichia, Acinetobacter,* and *Streptococcus* in *A. hebraeum* ticks from sheep and goats, which has significant implications for both veterinary and public health. It should be noted that 16S rRNA sequencing detects bacterial DNA but does not confirm active infection. However, the presence of these genera in tick microbiomes is concerning and warrants further investigation (e.g., targeted PCR confirmation), as their potential impact on livestock health and farm productivity could be substantial [38]. For instance, *Coxiella burnetii* has been associated with reproductive issues in ruminants, including abortions and stillbirths, which can have devastating effects on livestock populations [39, 40]. Additionally, pathogens like *Staphylococcus aureus*, *Streptococcus dysgalactiae*, and *Corynebacterium bovis* have been reported to cause intramammary infections, leading to mastitis and reduced milk production in cattle [41]. Moreover, emerging zoonotic pathogens like *Acinetobacter* species have been reported in various animals and food sources, posing potential public health risks [42, 43].

Venn diagram analyses based on bacterial ASVs provided insights into shared and unique microbial communities across hosts and sampling sites. Identifying a core microbiome, consisting of 200 bacterial ASVs, was shared across all locations, suggesting a stable and resilient microbial community maintained despite geographic variations. This finding aligns with previous studies emphasizing the importance of core microbiomes in tick species, which can influence their biology and interactions with pathogens [44–46]. The persistence of this core microbiome may play a critical role in the ticks' ecological fitness and capacity to act as vectors for tick-borne diseases.

Further analysis on geographic origin indicates localized microbial adaptations or interactions that could be influenced by environmental factors or host availability in the different regions. This observation is consistent with research suggesting that environmental conditions and host interactions significantly shape the microbiome composition in ticks [47, 48]. The unique bacterial ASVs identified in each location highlighted the tick microbiome's ecological diversity and adaptability. Such diversity may reflect the specific environmental niches and host interactions in each area, supporting that geographic and ecological factors are crucial in determining microbial community structure [49, 50].

Moreover, the high number of unique bacterial ASVs in Rooigrond suggests that this location may harbour a vibrant and diverse microbial community, potentially due to specific environmental conditions or a higher diversity of host species. This finding aligns with studies showing how habitat and host diversity can significantly influence the composition of tick microbiomes [51, 52]. Particularly, the presence and relative abundance of *Rickettsia* in *A. hebraeum* ticks in this study may also be linked to specific environmental conditions that favour tick survival and reproduction and the prevalence of associated pathogens. Differences in habitat between sheep and goats may contribute to variations in *A. hebraeum* ticks, *Rickettsia* prevalence, and other microbiota, consistent with research highlighting that tick species distribution and their associated pathogens can vary significantly based on geographic and climatic factors [53, 54]. Understanding these unique microbial communities is essential, as they may contribute to the ticks' vector competence and their interactions with pathogens, which could have implications for the transmission dynamics of tick-borne diseases [55, 56].

It is important to note that this study relies on 16S rRNA gene sequencing, which may limit taxonomic resolution. For instance, some *Rickettsia* species could not be identified at the species level. Additionally, pooling ticks may have concealed individual variations among hosts or locations. Future research that utilizes targeted PCR for pathogen confirmation and functional studies on tick microbiome interactions will be essential to validate and expand upon these findings. While this study highlights host species and geographic location as essential drivers of microbial community variation in ticks, other ecological factors such as tick sex and engorgement status were not evaluated in this study. This limitation stems from the available sample design and pooling strategy, which did not permit stratified comparisons. As these biological factors are known to influence microbial diversity and pathogen carriage, future studies with larger, stratified sample sizes are required to fully disentangle the relative contributions of host biology, vector biology, and geography in shaping tick-associated microbiomes.

## Conclusion

This study unveils critical host-driven shifts in the bacterial diversity of *A. hebraeum* ticks parasitising sheep and goats in Mahikeng, South Africa, offering insights into the complex microbial ecology of ticks in small-stock systems. Ticks from sheep harboured significantly more diverse and evenly balanced bacterial communities than goats, as confirmed by the Shannon index (*p* = 0.018) and the Simpson index (*p* = 0.02) diversity indices. In contrast, total observed species richness was comparable across hosts, indicating no significant difference in the number of bacterial taxa present. This suggests that host biology strongly shapes tick microbial community structure, as notable compositional differences emerged between ticks from different hosts. Beta diversity patterns showed that the tick bacterial communities clustered distinctly by host species. Sheep-associated ticks had a uniform bacterial community, while goat-associated ticks displayed higher variability, pointing to host biology and possibly environmental exposure as key microbial influencers. At the phylum level, Proteobacteria led the bacterial landscape across both hosts, an expected but critical finding, reinforcing its central role in tick biology. However, at finer taxonomic scales, goat-derived ticks were overwhelmingly dominated by *Rickettsiaceae* and *Rickettsia* spp. (over 50% abundance), which is a cause for concern regarding rickettsial disease transmission to both livestock and humans. Ticks from both hosts carry an a range of pathogens: *Coxiella*, *Bacillus*, *Staphylococcus*, *Corynebacterium*, *Ehrlichia*, *Acinetobacter*, and *Streptococcus*, many of which are implicated in reproductive losses, mastitis, zoonotic infections, and emerging antimicrobial resistance. Their detection in *A. hebraeum* warrants urgent attention from veterinary clinicians and public health stakeholders. A resilient core microbiome of 200 genera was detected across all locations, suggesting ecological stability. Distinct location-specific taxa, particularly the rich microbial diversity in Rooigrond, highlight how environmental factors, habitat, and host diversity shape tick microbiota and, consequently, vector competence. Overall, our study provides a baseline for tick microbiome composition in South African small stock systems and highlights host-associated differences that could influence pathogen dynamics. Future research should explore the functional roles of these microbiome members and how interventions might exploit these insights for tick-borne disease control.

## Supplementary Information

Below is the link to the electronic supplementary material.

ESM 1 DOCX (1.19 MB)

## Data Availability

The GenBank accession numbers for the 16S *rRNA* gene sequences generated from *Amblyomma hebraeum* specimens are: PP80989, PP809810, PP809812, PP809813, PP809811, PP809814, PP809815, PP809816, PP809817, PP809818, PP809824, and PP809819. The corresponding accession numbers for the 18S *rRNA* gene sequences from the same tick species are: PP826352, PP830056, PP830059, PP830060, PP830061, PP830062, PP830064, PP842196, and PP830071. Raw reads used in the present study were deposited to the National Center of Biotechnology Information (NCBI) Sequence Read Archive (SRA) database under BioProject ID PRJNA1246162 ([http://www.ncbi.nlm.nih.gov/bioproject/1246162](http://www.ncbi.nlm.nih.gov/bioproject/1246162)).
