# Supplementary material for: Bacterial Communities Harboured by Amblyomma Hebraeum Infesting Small Stock in Mahikeng city, South Africa
Source: Microb Ecol. 2025 Nov 8;88:118. doi: 10.1007/s00248-025-02630-0 (PMC12594692; doi:10.1007/s00248-025-02630-0)
Supplement: Supplementary file 1 [file 248_2025_2630_MOESM1_ESM.docx]

**Supplementary**

# **Bacterial communities harboured by *Amblyomma hebraeum* infesting small stock in Mahikeng city, South Africa**

Kealeboga Mileng^1*^, Sinalo Mani^2^, Jaco Bezuidenhout^1^, Prudent Mokgokong^1^, Tsepo Ramatla^1,3^, Oriel Thekisoe^1^, Kgaugelo E, Lekota^1^

^1^Unit for Environmental Sciences and Management, North-West University, Potchefstroom 2520, South Africa.

^2^Gastrointestinal Microbiology and Biotechnology, Agricultural Research Council Animal Production, Private Bag X2, Irene 0062, South Africa.

^3^Centre for Applied Food Safety and Biotechnology, Department of Life Sciences, Central University of Technology, 1 Park Road, Bloemfontein, 9300, South Africa.

*Corresponding author

Kealeboga Mileng, PhD

Email: [k.mileng@gmail.com](mailto:k.mileng@gmail.com)

**Supplementary Figure S1**. Alpha-diversity rarefaction curve showing the sequencing depth and microbial richness associated with Amblyomma hebraeum ticks from sheep and goats based on the 16S rRNA amplicon. Curves represent species richness (y-axis) as a function of sequencing depth (x-axis). Tick samples from goats (red) and tick samples from sheep (teal).

**Supplementary Figure S2.** Alpha diversity analysis of the microbial communities associated with *Amblyomma* *hebraeum* ticks was evaluated across four collection sites (Dihatshwane, Masutlhe, Ramatlabama, and Rooigrond). (A) Chao1, (B) Observed OTUs, (C) Shannon diversity index and (D) Simpson diversias evaluated across four collection sites index richness estimator.There were no significant differences observed across four collection sites (all p > 0.05).

**Supplementary Figure S3. A**nalysis of similarities (ANOSIM) of bacterial community structure in *Amblyomma hebraeum* ticks based on host species. The boxplot compares the rank-based dissimilarities of bacterial communities within the same host species (Goat or Sheep) to the dissimilarities between different host species (Between). The central line in each box represents the median rank, the box shows the interquartile range (IQR), and the whiskers extend to 1.5*IQR. A lower median rank within host groups indicates greater similarity. The analysis revealed a statistically significant, though weak, separation of microbial communities by host species (R = 0.163, p = 0.014).

**Supplementary Figure S4.** Analysis of similarities (ANOSIM) assessing the effect of geographical location on bacterial community structure in *Amblyomma hebraeum* ticks. The boxplot illustrates the rank-based dissimilarities between bacterial communities within the same village (Within) and between different villages (Between). The central line in each box represents the median rank, the box boundaries show the interquartile range (IQR), and the whiskers extend to 1.5*IQR. The analysis confirmed a statistically significant, though weak, separation of microbial communities by sampling location (R = 0.107, p = 0.047).

**Supplementary Figure S5.** Stacked bar plot representing the relative abundance of bacterial families in Amblyomma hebraeum ticks from (A) goats N=15 and (B) sheepN= 15 pools.
